# The Interactions of T Cells with Myeloid-Derived Suppressor Cells in Peripheral Blood Stem Cell Grafts

**DOI:** 10.3390/cells13181545

**Published:** 2024-09-14

**Authors:** Qingdong Guan, Scott G. Gilpin, James Doerksen, Lauren Bath, Tracy Lam, Yun Li, Pascal Lambert, Donna A. Wall

**Affiliations:** 1 Manitoba Blood and Marrow Transplant Program, Departments of Pediatrics and Child Health and Internal Medicine, University of Manitoba, Winnipeg, MB R3T 2N2, Canadadonna.wall@sickkids.ca (D.A.W.); 2 Department of Immunology, University of Manitoba, Winnipeg, MB R3T 2N2, Canada; 3 Manitoba Center for Advanced Cell and Tissue Therapy, Winnipeg, MB R3A 1R9, Canada; 4 Paul Albreachtsen Research Institute, CancerCare Manitoba, Winnipeg, MB R3A 1R9, Canada; 5 Department of Epidemiology and Cancer Registry, CancerCare Manitoba, Winnipeg, MB R3A 1R9, Canada; plambert@cancercare.mb.ca

**Keywords:** hematopoietic stem cell transplantation, peripheral blood stem cell graft, myeloid-derived suppressor cells, T lymphocytes, inhibitory receptors, allograft, autograft

## Abstract

The interaction of myeloid-derived suppressor cells (MDSCs) with T cells within G-CSF-mobilized peripheral blood stem cell (PBSC) grafts in patients undergoing autologous or allogeneic hematopoietic stem cell transplantation remains to be elucidated. Through studying allo- and auto-PBSC grafts, we observed grafts containing large numbers of T cells and MDSCs with intergraft variability in their percentage and number. T cells from autologous grafts compared to allografts expressed relative higher percentages of inhibitory receptors PD-1, CTLA-4, TIM-3, LAG-3, TIGIT and BTLA. Autograft T cells had decreased cell proliferation and IFN-γ secretion, which supported the possible presence of T cell exhaustion. On the contrary, graft monocytic MDSCs (M-MDSCs) expressed multiple inhibitory receptor ligands, including PD-L1, CD86, Galectin-9, HVEM and CD155. The expression of inhibitory receptor ligands on M-MDSCs was correlated with their corresponding inhibitory receptors on T cells in the grafts. Isolated M-MDSCs had the ability to suppress T cell proliferation and IFN-γ secretion and/or promote Treg expansion. Blocking the PD-L1-PD-1 signaling pathway partially reversed the functions of M-MDSCs. Taken together, our data indicated that T cells and M-MDSCs in PBSC grafts express complementary inhibitory receptor–ligand pairing, which may impact the quality of immune recovery and clinical outcome post transplantation.

## 1. Introduction

Granulocyte colony stimulating factor (G-CSF)-mobilized peripheral blood stem cells (PBSCs) are the most commonly used graft source for hematopoietic stem cell transplantation (HSCT) [1,2]. Conventionally, the PBSC graft is measured and dosed solely by CD34^+^ stem cell content. The CD34 graft component is a small fraction of the cells with the rest of the cellular content being lymphocytes, mature and immature myeloid and granulocytic cells, and other immune cells. The large numbers of graft passenger cells have the capacity to function in the early post-transplant period which is inflammatory following treatment with chemotherapy and radiation [3].

Myeloid cells, including conventional myeloid cells such as activated monocytes and neutrophils, and regulatory myeloid cells, such as myeloid-derived suppressor cells (MDSCs), are one of the first cell types that recover post HSCT [4]. In humans, the phenotypes of MDSCs are CD33^+^CD15^−^CD14^+^HLA-DR^−/low^ monocytic MDSCs (M-MDSCs), CD33^+^CD14^−^CD11b^+^CD15^+^ polymorphonuclear MDSCs (PMN-MDSCs) and CD33^+^CD14^−^CD15^−^HLA-DR^−^ early-stage MDSCs (e-MDSCs) [5,6]. MDSCs have the ability to suppress both innate and adaptive immune responses and promote immune regulatory cells—all central elements in the processes of graft-vs.-leukemia effects and graft-vs.-host disease (GVHD) [7,8,9]. Several studies suggest that MDSCs in allo-PBSCs can suppress T cell function and predict acute GVHD [10,11]. The infusion of fewer CD14^+^HLA-DR^lo^ M-MDSCs or CD11b^+^HLA-DR^lo^Lineage^−^ MDSCs in an autograft improves the overall survival and progression-free survival post auto-HSCT [12,13]. However, there was no association between bone marrow M-MDSCs and outcome post allo-HSCT in one study, but this has not been studied in depth [14].

Dysregulated T cell responses including T cell exhaustion play an important role in the pathogenesis and progression of cancers [15,16]. A clinical correlation between T cell exhaustion in the bone marrow/blood and relapse post allo-HSCT or auto-HSCT in patients with leukemia or multiple myeloma [17,18,19] and the fact that checkpoint inhibitors can reverse T cell exhaustion in patients with multiple myeloma have been reported [18,19]. Having a higher number of infused CD4^+^PD-1^−^ was associated with better progression-free survival post auto-HSCT in patients with non-Hodgkin lymphoma in one study [13]. The absolute and relative content of the passenger T and myeloid cells in the graft has been reported as being important. The ratio of lymphocytes to monocytes in autografts has been predictive of relapse and/or survival post auto-HSCT [20,21].

In the present study, we systematically studied the relationships between T cells and myeloid cells in grafts, with a focus on the expression patterns of T cell inhibitory receptor–ligands between T cells and M-MDSCs in PBSC allografts and autografts, and examined the function of T cells and MDSCs in the grafts.

## 2. Methods

### 2.1. Mobilization and Collection of PBSCs

We retrospectively studied grafts from G-CSF-mobilized healthy sibling/unrelated donors for use in allo-HSCT and grafts from patients with multiple myeloma or lymphoma undergoing autologous transplant. The grafts were collected between 2010 and 2014 at CancerCare Manitoba, and donor demographics are seen in Table 1. All donor samples were obtained after written informed consent, in accordance with the Declaration of Helsinki, and approved by the ethics committee of the University of Manitoba (HS16600 (H2013:306)). The allogeneic healthy donors received a 5-day course of 10 µg/kg per day subcutaneous filgrastim mobilization, while the autologous donors received combination chemotherapy and filgrastim mobilization.

### 2.2. Graft Analysis

CD34^+^ cell enumeration was determined on the day of collection by flow cytometry on FACSCanto II (BD Biosciences, Mississauga, ON, Canada) and analyzed by BD FACSDIVA software 8.0.1 as part of the clinical practice. CD34^+^ cell enumeration was performed according to the International Society of Hematotherapy and Graft Engineering guidelines [22]. For the detailed graft analysis, a frozen aliquot of the final PBSC products was stained with fluorescence-labeled antibodies and viability dye to evaluate the presence of myeloid cells (CD33^+^CD14^+^CD15^−^HLA-DR^−/low^ M-MDSC, CD33^+^CD14^−^CD15^+^ PMN-MDSC, CD33^+^CD14^−^CD15^−^HLA-DR^−/low^ e-MDSC and CD33^+^CD14^+^HLA-DR^hi^ conventional monocytes), T cells (CD3^+^T, CD4^+^T, CD8^+^T, CD3^+^CD56^−^TCRαβ^+^CD4^−^CD8^−^ double-negative regulatory T cells (DNTreg) and CD3^+^CD4^+^CD25^+^Foxp3^+^Treg), CD3^−^CD56^+^NK, CD3^+^CD56^+^NKT and CD19^+^B cells. To study the subpopulations of T cells, fluorescence-labeled antibodies against CD45RA and CCR7 were added into the T cell staining panel [23]. CD45RA^+^CCR7^+^ were considered as naïve T cells, CD45RA^+^CCR7^−^ as effector T cells, CD45RA^−^CCR7^−^ as effector memory T cells and CD45RA^−^CCR7^+^ as central memory T cells. To study the inhibitory receptors on T cells, fluorescence-labeled antibodies against PD-1, CTLA-4, TIM-3, LAG-3, TIGIT and BTLA were added into the T cell staining panel. To evaluate the expression of ligands of inhibitory receptors, fluorescence-labeled antibodies against PD-L1, CD86, CD155, HVEM and Galectin-9 were added into the MDSC staining panel. For intracellular staining, after surface staining, cells were fixed and permeabilized with the Transcription Factor Staining Buffer Set (eBioscience, San Diego, CA, USA) or Cytofix/Cytoperm Fixation/Permeabilization kit (BD Biosciences) and stained with the relevant antibodies. An analysis was performed on FACSCanto II and Flowjo software 10.0.

### 2.3. T Cell Proliferation and Cytokine Secretion from Graft Cells

CD3^+^ T cells were sorted from the grafts using a CD3 negative selection kit according to the manufacturer’s protocol (StemCell Technologies, Vancouver, BC, Canada). The purity of CD3^+^T cells was confirmed by flow cytometry (purity > 90%). 1 × 10^5^ CD3^+^T cells were seeded into the 96-well plates in the complete DMEM culture media with recombinant human IL-2 (2 µg/mL) and incubated for 4–5 days at 37 °C and 5% CO_2_. Cells were stimulated with PMA/ionomycin/BFA (Cell Stimulation Cocktail, eBioscience) for 4 h. Then, cells were harvested to stain CD3, Ki67 and IFN-γ according to the manufacturer’s protocol. An analysis was performed on FACSCanto II and Flowjo software 10.0.

### 2.4. Effects of MDSC Subsets on the Proliferation and Expansion of T Cells

To evaluate the function of MDSCs, M-MDSCs and PMN-MDSCs were enriched by a positive selection from fresh PBSC grafts based on surface markers (CD33^+^CD15^−^CD14^+^HLA-DR^−/low^ for M-MDSCs, CD33^+^CD14^−^CD15^+^ for PMN-MDSCs) using the MoFlo XDP cell sorter (Beckman Coulter, Mississauga, ON, Canada) [6]. The purity of M-MDSCs and PMN-MDSCs was confirmed by flow cytometry (purity > 95%). The morphology of M-MDSCs was examined by HEMA-3 staining as we described previously [6,24]. The suppressive effect of MDSC subsets on lymphocyte proliferation and T cell differentiation were evaluated as we described previously [4,6,25]. Briefly, CD3^+^T cells were sorted from third-party healthy donor PBMCs or autografts using a CD3 negative selection kit (Stemcell Technologies, Vancouver, BC, Canada). Then, 1 × 10^5^ CD3^+^T cells were stimulated with anti-CD3/CD28 microbeads (1.8 µL/well, Life Technologies, Carlsbad, USA) in the presence of M-MDSCs or PMN-MDSCs at the ratios of CD3^+^T: MDSC (1:0.8 and 1:0.4) in 96-well plates for 4–5 days. T cell proliferation was evaluated by CFSE or Ki67 staining. Cells were stained with fluorescence-labeled antibodies against CD4, CD8 and/or IFN-γ for detecting T cell differentiation. In some experiments, a fluorescence-labeled antibody against PD-1 was also used to stain T cells after co-culturing M-MDSCs with T cells to evaluate whether M-MDSCs can increase PD-1 expression in T cells. To block the interaction of PD-L1 with PD-1, anti-PD-L1 (10 μg/mL) were added into the T cell/M-MDSC co-culture system at the beginning of co-culture. To evaluate the effects of MDSCs on the expansion of Treg, CD3^+^T cells were co-cultured with MDSCs at different ratios for 4–5 days. Cells were then stained with CD4, CD25 and Foxp3 for detecting Treg according to the manufacturer’s instruction. An analysis was performed on FACSCanto II and FlowJo software. Cell counts were performed using the COULTER^®^ AcT diff™ Analyzer (Beckman Counter, Mississauga, ON, Canada). The suppression percentage of MDSCs on T cell proliferation was calculated as 100%—(proliferated T cell number of MDSC group/proliferated T cell number of control group × 100%) [25].

### 2.5. Statistical Analysis

Values were expressed as mean ± SD when normally distributed and as violin plot when not normally distributed. ANOVA followed by Newman–Keuls multiple comparison test was used to compare cell values in different grafts when normally distributed, and the Kruskal–Wallis test followed by Dunn’s multiple comparison test when not normally distributed. Spearman correlations were used to assess the relationship among different cell groups in the graft, and two-sided *p*-values from this correlation analysis were corrected for multiple comparisons by using the Benjamani–Hochberg False Discovery Rate (FDR) procedure at an FDR-adjusted *p* < 0.05.

## 3. Results

### 3.1. Variation between Individual Grafts and between Different Graft Sources

**Figure 1:**
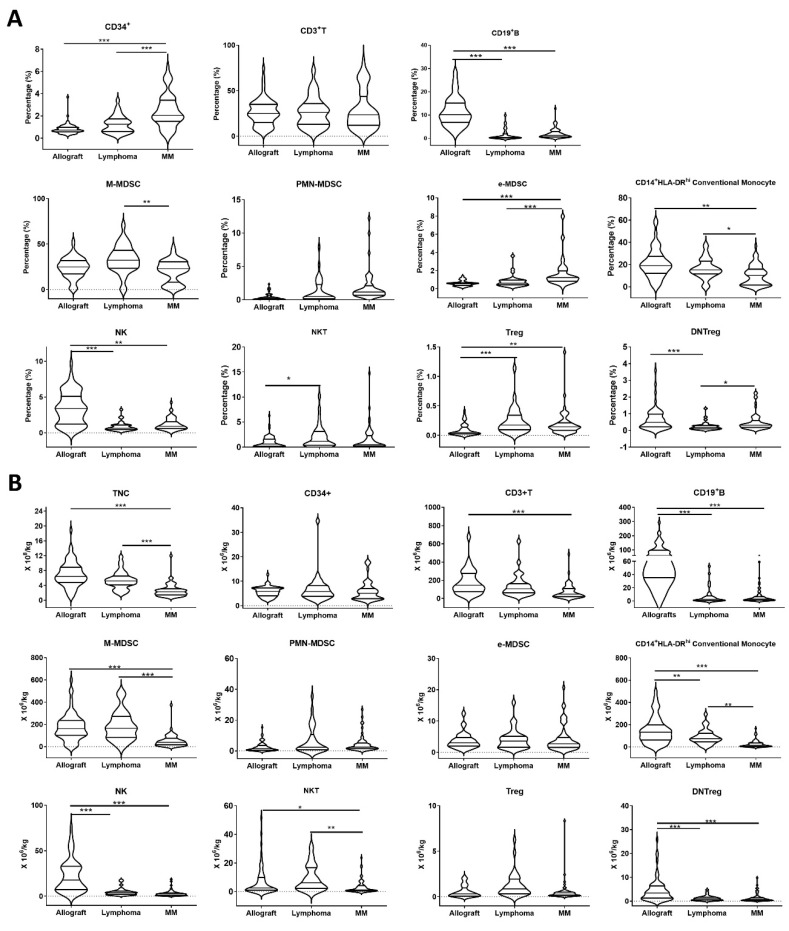
A comparison of cellular compositions in allografts, lymphoma autografts and MM autografts. The percentages of CD34^+^ stem cells, CD33^+^CD14^+^CD15^−^HLA-DR^−/low^ M-MDSC, CD33^+^CD14^−^CD15^+^ PMN-MDSC, CD33^+^CD14^−^CD15^−^HLA-DR^−^ e-MDSC, CD3^+^T, CD3^−^CD56^+^NK, CD3^+^CD56^+^NKT, CD3^+^CD56^−^TCRαβ^+^CD4^−^CD8^−^ DNTreg, CD3^+^CD4^+^CD25^+^Foxp3^+^Treg, CD19^+^B cells, and CD14^+^HLA-DR^hi^ conventional monocytes in the grafts were evaluated by staining the graft cells using fluorescence labeled antibodies and analyzed by Flowjo software. (**A**) Percentages of cell subsets in the grafts. (**B**) Infusion dose of cell subsets in the grafts. *, *p* < 0.05; **, *p* < 0.01; ***, *p* < 0.001.

The levels of immune cell subpopulations varied between individual grafts and between different graft sources. As expected, less than 2% of the PBSC graft was CD34^+^ stem cells (Figure 1A), which determined the infusion cell dose for transplant. The rest of the infused graft was cells of lymphoid and myeloid lineage, such as T cells, MDSCs, monocytes, B cells, NK cells, NKT cells and regulatory T cells.

Approximately half (41.2–55.4%) of passenger cells in the grafts were CD33^+^ myeloid lineage, such as CD33^+^CD14^+^HLA-DR^hi^ conventional monocytes and MDSCs. M-MDSCs were a major subset of MDSCs in the grafts, with an average percentage 20.6–33.3%, but showing a wide intergraft variability ranging from 0 to 70.6% of the infused graft cells; however, the percentages of PMN-MDSCs and e-MDSCs accounted for less than 2.0% in the cryopreserved grafts (Figure 1A).

The other major cellular component of the graft was lymphoid cells. The CD3^+^T cell average percentage was 20.4–28.4%, with over 90% of T cells being conventional TCRαβ^+^CD3^+^ T cells. Treg, double-negative Treg (DNTreg), CD19^+^B cell, CD3^−^CD56^+^NK cell and CD3^+^CD56^+^NKT populations also showed varied percentages in the grafts (Figure 1A), with allografts having higher percentages of NK, B cells and DNTreg and autografts having higher percentage of Treg.

Infused allografts usually had higher doses of CD19^+^B cells, NK cells, DNTreg and conventional monocytes compared to the infused autografts (Figure 1B), which were similar to their percentages.

### 3.2. Characterization of T Cells in the Grafts

**Figure 2:**
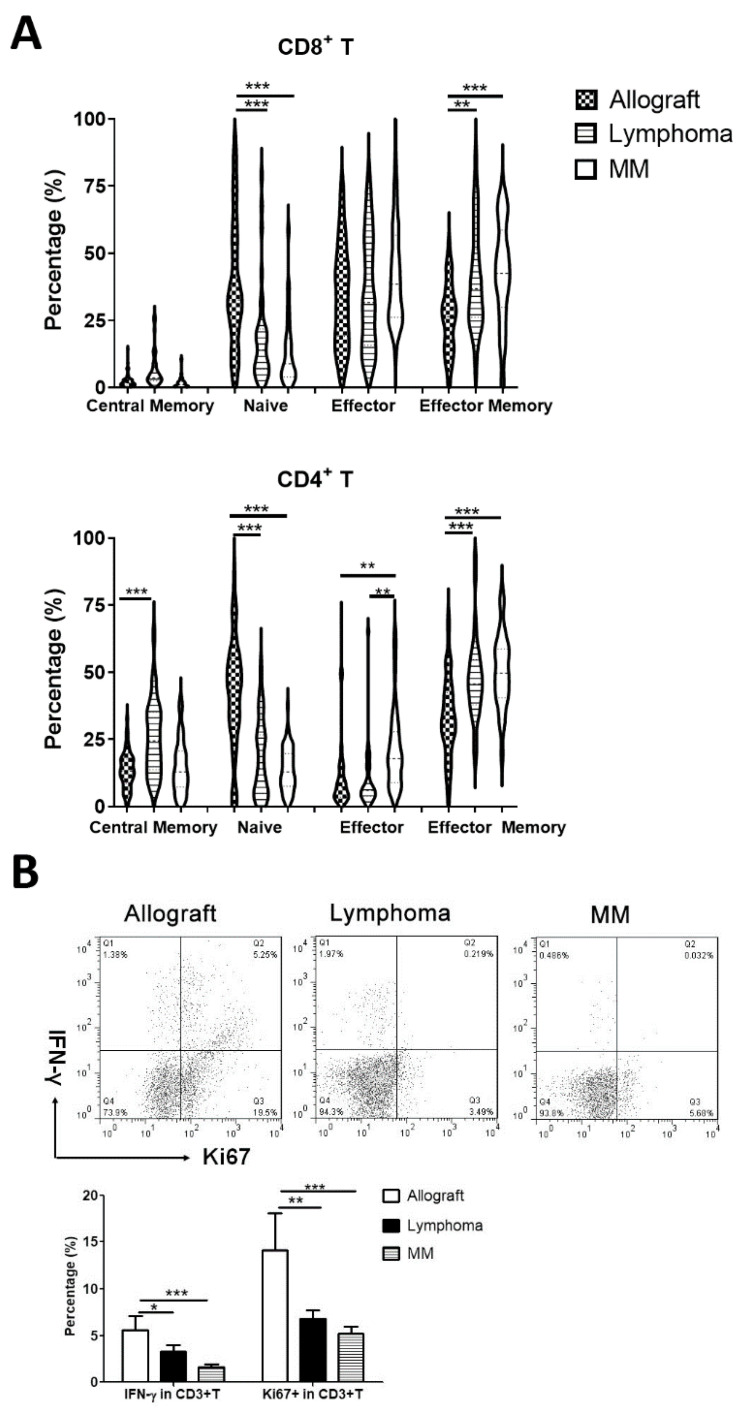

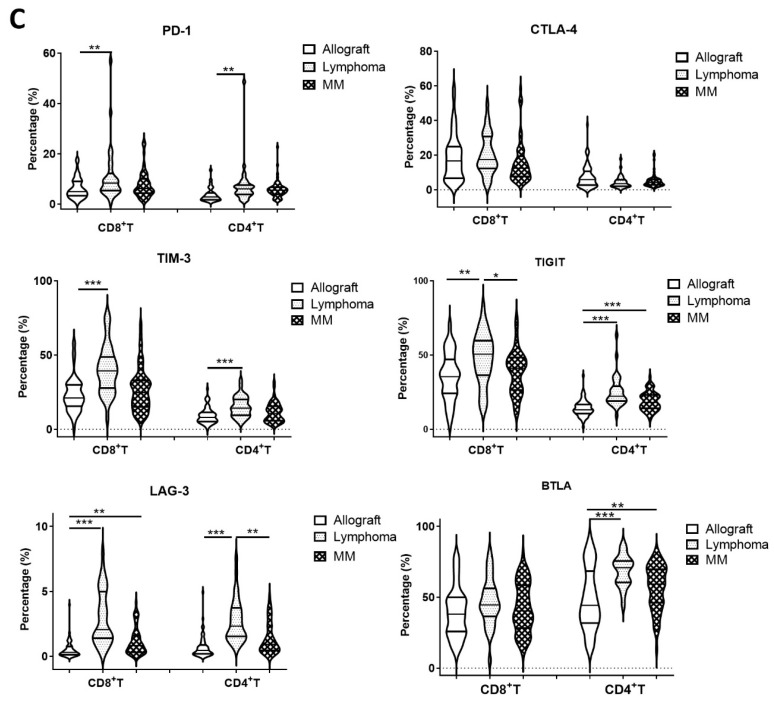
Characterization of T cells in the grafts. To evaluate the percentages of naïve, effector or memory T cells, fluorescence-labeled antibodies against CD45RA and CCR7 were added into the staining panel. To evaluate the proliferation and cytokine secretion of T cells in the grafts, sorted CD3^+^T cells from allografts and autografts were cultured for 5 days and then were stained with Ki67 to evaluate T cell proliferation, with fluorescence-labeled anti-IFN-γ for cytokine secretion. The expression of inhibitory receptors PD-1, CTLA-4, TIM-3, LAG-3, TIGIT and BTLA on CD4^+^T and CD8^+^T cells were evaluated by staining the graft cells using fluorescence-labeled antibodies. (**A**) Percentages of naïve, effector, central memory and effector memory T cells in the grafts. (**B**) The expression levels of Ki67 and IFN-γ in CD3^+^T cells of 10 allografts, 9 lymphoma autografts and 10 MM autografts. (**C**) The expression pattern of inhibitory receptors on T cells in the grafts. *, *p* < 0.05; **, *p* < 0.01; ***, *p* < 0.001.

Through staining CD45RA and CCR7 [23], we studied the percentage of CD45RA^+^CCR7^+^-naïve, CD45RA^−^CCR7^+^ central memory T, CD45RA^−^CCR7^−^ effector memory T and CD45RA^+^CCR7^−^ effector T cells in the graft. This showed that allografts had higher percentages of naïve CD4^+^ and CD8^+^T cells, while autografts had more effector memory CD4^+^T and CD8^+^T cells (Figure 2A).

T cell exhaustion is characterized by poor proliferation, decreased cytokine secretion and increased inhibitory receptor expression. We first evaluated the proliferation and cytokine secretion of CD3^+^ T lymphocytes in the allografts and lymphoma and MM autografts. CD3^+^T cells in lymphoma and MM autografts had a lower expression of the proliferation marker Ki67 and cytokine IFN-γ compared to CD3^+^T cells in allografts, supporting the idea that CD3^+^T cells in the autograft were less able to proliferate or secrete cytokines (Figure 2B).

We further evaluated the expression of inhibitory receptors (PD-1, CTLA-4, TIM-3, LAG-3, TIGIT and BTLA) on T cells in the grafts. The graft CD4^+^T and CD8^+^T cell expression of inhibitory receptors was variable (Figure 2C). Autograft T cells expressed higher levels of inhibitory receptors than allograft T cells. These results indicated the possible presence of T cell exhaustion in autografts.

### 3.3. M-MDSCs Expressed Multiple Ligands of Inhibitory Receptors at Variable Levels and Had Immune Regulatory Function

**Figure 3:**
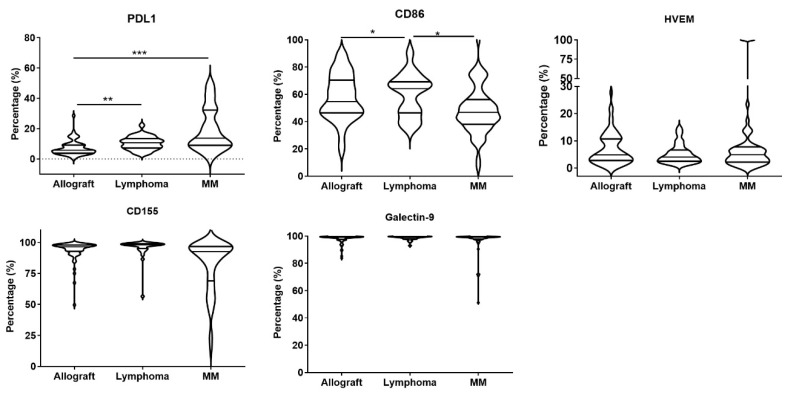
The expression of inhibitory receptor ligands on M-MDSCs in the grafts. The expression of inhibitory receptor ligands PD-L1, CD86, Galectin-9, CD155 and HVEM were evaluated by staining the graft cells using fluorescence-labeled antibodies. Analysis was gated on M-MDSCs. *, *p* < 0.05; **, *p* < 0.01; ***, *p* < 0.001.

The inhibitory receptors expressed on T cells, interacting with their corresponding ligands expressed on antigen-presenting cells or tumor cells, such as PD-1/PD-L1, CTLA-4/CD86, TIM-3/Galectin-9, TIGIT/CD155, and BTLA/HVEM, play important roles in regulating T cell functions [15]. As M-MDSCs are the major MDSC subset in cryopreserved PBSC grafts, we examined the expression of inhibitory receptor ligands on M-MDSCs. M-MDSCs expressed the inhibitory receptor ligands PD-L1 and CD86 with high variability, ranging from 0 to 38% and 17.5 to 91.8%, respectively (Figure 3). The expression of HVEM in M-MDSCs ranged from 0 to 23%, except for a subset of MM autografts which had a 100% expression of HVEM. Galactin-9 and CD155 were constitutively expressed in M-MDSCs in allografts and autografts, with the exception of several MM autografts showing M-MDSCs with a low expression of galectin-9 and CD155.

We further analyzed the relationship between the expression of inhibitory receptors on T cells with their corresponding ligands on M-MDSCs in the grafts. As shown in Table 2, the percentage of CD86^+^M-MDSCs was inversely correlated with the percentages of CTLA-4^+^CD4^+^T and CTLA-4^+^CD8^+^T cells in the grafts. The percentage of PD-L1^+^M-MDSCs was inversely correlated with the percentage of PD-1^+^CD4^+^T and PD-1^+^CD8^+^T in autografts, and the percentage of HVEM^+^M-MDSCs was inversely correlated with BTLA^+^CD4^+^T and BTLA^+^CD8^+^T in allografts and MM autografts.

**Figure 4:**
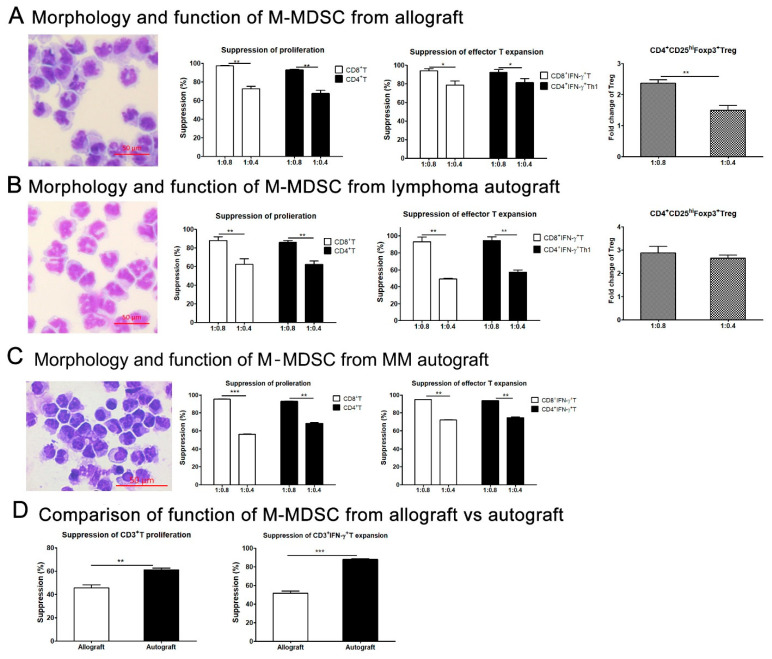
Morphology and immune regulatory function of M-MDSCs in the allografts, lymphoma and MM autografts. M-MDSCs were sorted from allografts, lymphoma and MM autografts using flow cytometer based on cell surface markers. The morphology of M-MDSCs was evaluated by HEMA-3 staining and then read via microscopy (200× magnification). By using CD3 negative selection kit, CD3^+^T cells were sorted from peripheral blood of third-party healthy donors or from PBSCs grafts of lymphoma or multiple myeloma patients. CD3^+^T cells were co-cultured with M-MDSCs at different ratios in the presence of anti-CD3/CD28 for 4–5 days. T cell proliferation, IFN-γ secretion and Treg expansion were evaluated. (**A**) Morphology and immune regulatory function of M-MDSCs sorted from allograft on third-party T cell proliferation, IFN-γ secretion and CD4^+^CD25^+^Foxp3^+^Treg expansion. (**B**) Morphology and immune regulatory function of M-MDSCs sorted from lymphoma autograft on autologous T cell proliferation, IFN-γ secretion and autologous CD4^+^CD25^+^Foxp3^+^Treg expansion. (**C**) Morphology and immune regulatory function of M-MDSCs sorted from MM autograft on autologous T cell proliferation and IFN-γ secretion. (**D**) Comparison of suppressive function of M-MDSCs sorted from allograft or autograft against the same third-party healthy donor T cells at a ratio of T:M-MDSC (1:0.4). *, *p* < 0.05; **, *p* < 0.01; ***, *p* < 0.001. These results represent one of two to three independent experiments.

M-MDSCs sorted from fresh allo-PBSC grafts had immature monocyte morphology and suppressed allogeneic T cell proliferation and IFN-γ secretion, and drove allogeneic Treg expansion in vitro (Figure 4A). Similarly, M-MDSCs sorted from autografts showed a similar morphology, suppressed autologous T cell proliferation and cytokine production, and/or promoted autologous Treg expansion (Figure 4B,C). Interestingly, M-MDSCs sorted from autografts showed much stronger suppression than those from allografts against the same third-party T cells. This may be due to the fact that M-MDSCs in autografts were licensed by cytokines and/or growth factors released during tumor chemotherapy or by tumor cells [7,26,27].

**Figure 5:**
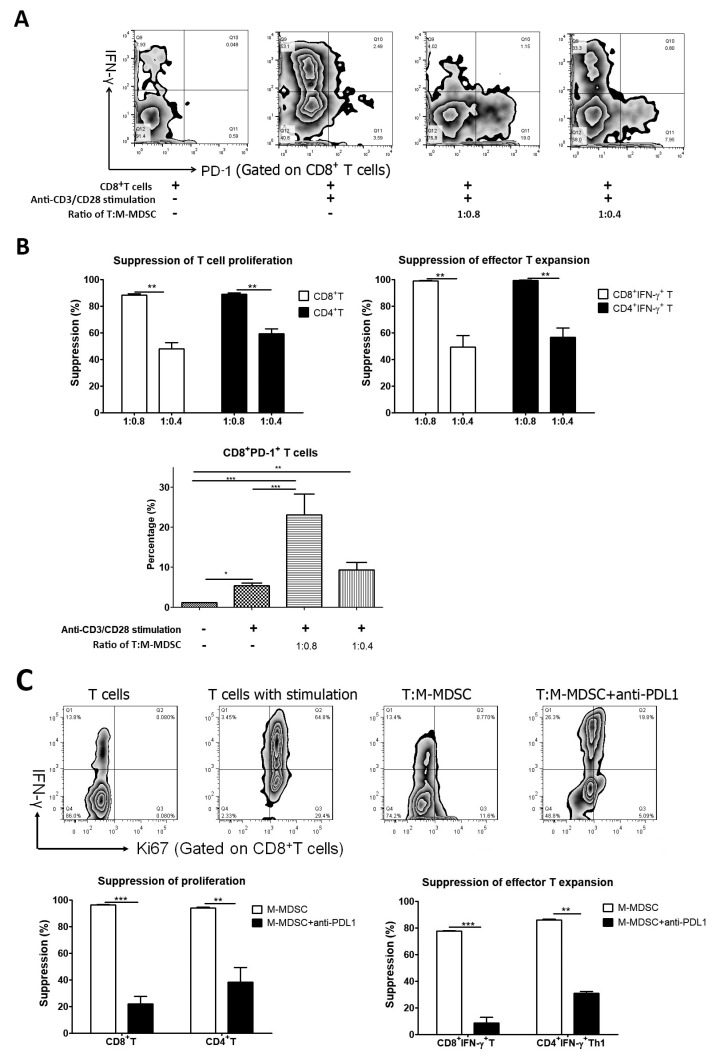
M-MDSCs drive CD8^+^T cell exhaustion. M-MDSCs sorted from lymphoma autograft were co-cultured with sorted autologous CD3^+^T cells in the presence of anti-CD3/CD28 for 4–5 days. The T cell proliferation, expression of PD-1 and IFN-γ on CD8^+^T cells and CD4^+^T cells were evaluated. To block the interaction of PD-L1 with PD-1, anti-PD-L1 (10 μg/mL) was added into the co-culture system at the beginning. (**A**) The representative figures of expression of PD-1 and IFN-γ on CD8^+^T cells. (**B**) Effects of M-MDSCs on autologous T cell proliferation, effector T expansion and PD-1 expression. (**C**) The effects of anti-PD-L1 on the suppressive function of M-MDSCs sorted from lymphoma autograft. *, *p* < 0.05; **, *p* < 0.01; ***, *p* < 0.001.

Further, it showed that M-MDSCs isolated from the lymphoma autograft dramatically increased PD-1 expression on autologous CD8^+^T cells in the cell co-culture in a dose-dependent manner whilst also suppressing CD8^+^T cell proliferation and cytokine secretion (Figure 5A,B). The addition of anti-PD-L1 in the cell co-culture reduced the suppression of M-MDSCs on CD8^+^T cell proliferation and effector CD8^+^IFN-γ^+^T cell differentiation (Figure 5C), which indicated that anti-PD-L1 could partially block the function of M-MDSCs. This raises the possibility of graft M-MDSCs driving autologous CD8^+^T cell exhaustion through the PD-1/PD-L1 pathway. M-MDSCs also suppressed the proliferation and IFN-γ secretion of autologous CD4^+^T cells, but M-MDSCs downregulated their expression of PD-1 (Supplementary Figure S1). We also evaluated whether M-MDSCs derived from allografts have the ability to drive T cell exhaustion. M-MDSCs derived from allo-PBSC graft decreased the expression of PD-1 on third-party T cells (Supplementary Figure S2), as well as suppressing T cell proliferation and IFN-γ secretion.

**Figure 6:**
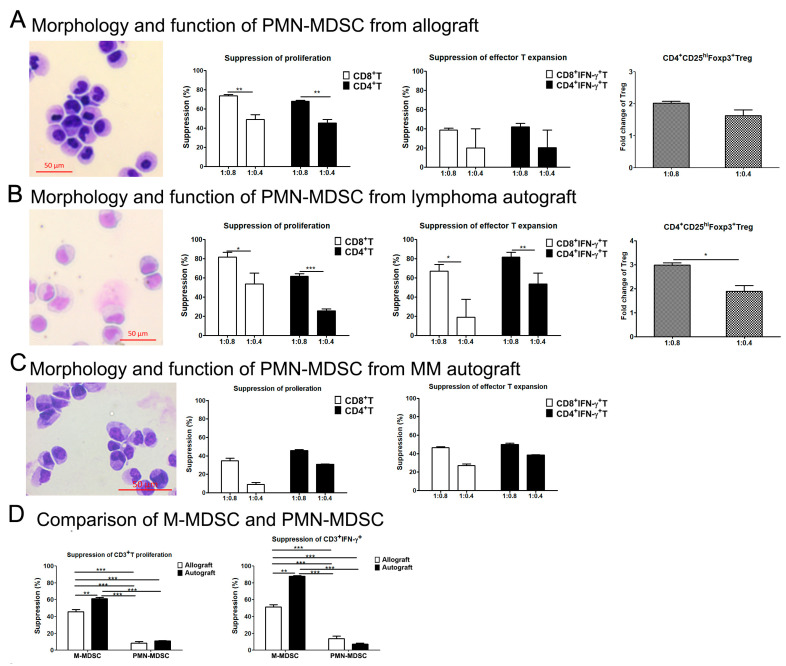
Morphology and immune regulatory function of PMN-MDSCs in the allografts, lymphoma and MM autografts. The morphology of sorted PMN-MDSCs was evaluated by HEMA-3 staining and then read via microscopy (200× magnification). The CD3^+^T cells sorted from healthy third-party donor or autologous donor were co-cultured with PMN-MDSCs at different ratios in the presence of anti-CD3/CD28 for 4–5 days. T cell proliferation, IFN-γ secretion and Treg expansion were evaluated. (**A**) Morphology and immune regulatory function of PMN-MDSCs sorted from allograft on third-party T cell proliferation, IFN-γ secretion and CD4^+^CD25^+^Foxp3^+^Treg expansion. (**B**) Morphology and immune regulatory function of PMN-MDSCs sorted from lymphoma autograft on T cell proliferation, IFN-γ secretion and CD4^+^CD25^+^Foxp3^+^Treg expansion. (**C**) Morphology and immune regulatory function of PMN-MDSCs sorted from MM autograft on T cell proliferation and IFN-γ secretion. (**D**) Comparison of the suppressive function of M-MDSCs and PMN-MDSCs from the same graft at a ratio of T:M-MDSC (1:0.4). These results represent one of two to three independent experiments. *, *p* < 0.05; **, *p* < 0.01; ***, *p* < 0.001.

Using the same method, we evaluated the morphology and function of PMN-MDSCs in the fresh allograft and autograft. Sorted PMN-MDSCs from allografts, lymphoma and MM autografts showed immature granulocyte morphology (Figure 6). PMN-MDSCs sorted from allograft, lymphoma and MM autografts showed the ability to suppress autologous T cell proliferation and IFN-γ secretion, and/or promote the expansion of Treg (Figure 6). However, the suppressive ability of PMN-MDSCs sorted from the grafts was less pronounced than that of M-MDSCs sorted from the same graft on the suppressing T cell proliferation and IFN-γ secretion (Figure 6D).

Taken together, these results suggested that the immune regulatory functional MDSC subsets were present in the PBSC grafts.

## 4. Discussion

While we focus on the CD34^+^ stem cells in the grafts used in allogeneic and autologous transplant, the grafts contain large numbers of immune cells that are capable of guiding immune reconstitution post-transplant. Here, we show that there are a range of regulatory and inflammatory lymphocytes expressing variable multiple inhibitory receptors, including PD-1, CTLA-4, TIM-3, LAG-3, TIGIT and BTLA. T cells in autografts were less proliferative and released less cytokines. This, combined with the expression of inhibitory receptors, supports the idea that the T cells infused at the time of autologous transplant might be exhausted. Additionally, graft-derived M-MDSCs express multiple inhibitory receptor ligands that are capable of interacting with T cell inhibitory receptors. The expression of the inhibitory receptors PD-1, CTLA-4 and BTLA on T cells was negatively correlated with the expression of their corresponding ligands PD-L1, CD86 and HVEM on M-MDSCs.

We noted that graft M-MDSCs had a variable expression of inhibitory receptor ligands PD-L1, CD86, Galectin-9, CD155 and HVEM (Figure 3). Isolated M-MDSCs and PMN-MDSCs showed strong immune suppressive functions, including inhibiting T cell proliferation, suppressing the expansion of CD4^+^Th1 and CD8^+^IFN-γ^+^T cells, and/or promoting CD4^+^CD25^hi^Foxp3^+^Treg in vitro. MDSCs may play important roles in relapse/GVHD/GVL post PBSC transplantation. Studies have shown that the infusion of allografts with a high number of M-MDSCs or PMN-MDSCs have a lower incidence of grade II-IV acute GVHD and/or extensive chronic GVHD [11,28], but have not seen a correlation of MDSCs in the allograft with relapse/overall survival post allo-HSCT [11]. Our previous report also showed that the rapid recovery of PMN-MDSCs or even higher PMN-MDSCs at 1 month post allo-HSCT had a lower risk to develop acute GVHD [4]. The high frequency of M-MDSCs in the peripheral blood at a median of 14 days after allo-HSCT was associated with a lower incidence of grade III-IV acute GVHD but with a high probability of relapse [29]. A high number of M-MDSCs in the peripheral blood before conditioning chemotherapy pre-HSCT was associated with a shorter time to progression after auto-HSCT for patients with multiple myeloma [30]. The infusion of fewer M-MDSCs into patients with NHL improved the overall survival and progression-free survival post auto-HSCT [12]. These results indicated that M-MDSCs may advance cancer progression/relapse post HSCT, which was contradictory to murine studies. In a murine model, MDSCs could preserve graft-versus-leukemia/tumor effects while attenuating GVHD, and it found that MDSCs functioned through regulating the Th17/Treg signaling pathway or inducing NKG2D^+^CD8^+^T cells [9,31,32]. In the future, we will evaluate the roles of MDSCs and their expressed ligands of inhibitory receptors in PBSC grafts with clinical outcomes post HSCT.

T cell exhaustion is a hyporesponsive state of T cells, with the loss of effector functions and expression of multiple inhibitory receptors [15,33]. The revitalization of exhausted T cells could reinvigorate immunity [15]. Checkpoint inhibitors targeting immune checkpoints, through blocking the interactions of inhibitory receptors on T cells with their ligands on tumor cells or other immune cells, have been widely used in clinics to treat multiple cancers [15,34,35,36]. T cell exhaustion has been noted in hematological malignancies and relapse post HSCT, with the blocking of these checkpoint restoring T cell function [6,18,19,37,38,39]. It is not clear whether HSCT graft T cell expression of exhaustion markers correlates with relapse, GVHD, or survival post HSCT. The work by Porrata et al., showed that the infusion of a higher number of CD4^+^PD-1^−^ was associated with better progression-free survival post auto-HSCT in patients with NHL [13]. Our present study showed that T cells in PBSC grafts—both from healthy donors and patients with lymphoma or multiple myeloma—expressed variable levels of inhibitory receptors PD-1, CTLA-4, TIM-3, LAG-3, TIGIT and BTLA on their surface, with T cells in autografts expressing a higher level of these inhibitory receptors (Figure 2). The expression levels of inhibitory receptors on T cells were inversely correlated with the expression of ligands of inhibitory receptors on M-MDSCs (Table 2). Graft T cells from patients with lymphoma and MM showed decreased cell proliferation and IFN-γ secretion when compared to T cells in allografts from healthy donors. These results indicated that T cell exhaustion may be present in the lymphoma and MM autografts. It suggests that checkpoint inhibitors such as anti-PD-L1 or CTLA-4 may be able to result in a less MDSC-driven suppressive immune phenotype and improved function of graft (and host) T cells early post-transplant—at a time when the tumor microenvironment has been disrupted by intensive chemo/radiation therapy. The early post-transplant period is marked by stress hematopoiesis by cells in the graft—which could be guided to be more immunosuppressive (for GVHD prophylaxis/treatment) or less immunosuppressive (to optimize the graft-vs-leukemia/lymphoma/tumor effect). Our study shows that large numbers of functional MDSCs and T cells are infused with the graft—in numbers that would be considered therapeutic products in their own right. Given the intergraft variability in both number and function, it may be worth broadening our characterization of grafts to include these potentially biologically active subsets.

T cell exhaustion is driven by many factors, including persistent antigen stimulation and the tumor environment [15,33]. It is not clear whether MDSCs drive T cell exhaustion in an HSCT setting. There is growing evidence that MDSCs can drive T cell exhaustion in tumor models and HBV infection models [40,41]. MDSCs promoted murine breast cancer progression by activating T cell exhaustion and inducing an epithelial–mesenchymal transition [40]. Blocking PD-L1 on MDSCs abrogated immune suppression and improved tumor control in a murine model of ovarian cancer [42]. In this study, we showed that co-culture of MDSCs with T cells led to lower T cell proliferation and lower IFN-γ secretion. Also, M-MDSCs from lymphoma autografts upregulated the expression of inhibitory receptor PD-1 on CD8^+^T cells when co-culturing M-MDSCs with autologous T cells (Figure 5). When the interaction of PD-L1 on M-MDSCs with PD-1 on T cells was blocked with anti-PD-L1, there was a partial reversal of the suppressive function of M-MDSCs. These results support the idea that M-MDSCs may drive CD8^+^T cell exhaustion in a lymphoma HSCT setting via the PD-L1/PD-1 pathway.

Due to the retrospective nature of this study, the enumeration of immune cell subsets in the allografts have been only performed on cryopreserved cryovials. This could represent a limitation of our study. MDSCs, especially PMN-MDSCs, do not survive cryopreservation/thaw processes well [43]. This is not a limitation for autologous HSCTs since they are all performed with cryopreserved products. Since allografts are generally delivered fresh without cryopreservation, there may in fact be greater numbers of MDSCs infused. In the future, we will start a prospective study to evaluate the effects of MDSCs in fresh allografts. The other limitation of this study was that we had limited numbers of allo-/auto-PBSC graft samples being examined, so we could not analyze the effects of infused T cells and M-MDSCs in the grafts on the clinical outcome post HSCT.

While it is conventional to collect allogeneic grafts following G-CSF mobilization and autologous grafts following G-CSF ± chemotherapy recovery, we have not looked at pericollection variables that will impact the immunologic state of the nonhematopoietic cells collected in the graft—recent inflammation, the timing of collection with respect to the intensity of prior chemotherapy, and the impact of G-CSF exposure on both myeloid and T cells in the graft. The grafts are typically infused post chemotherapy/radiation conditioning therapy—and the infused cells are exposed to tissue damage, inflammatory signals, and ideally the disruption of the tumor microenvironment. Here, we show the variability and functional potential of immune cells contained in the graft and suggest that it might be worth characterizing PBSC grafts, not only based on CD34^+^ stem cells but also on their other cell contents. As the numbers of MDSCs and T cells contained in the graft are in the range of a cellular therapy product, they may have a functional role and potentially targeted to modulate post-transplant immune function [44].

## Figures and Tables

**Table 1:** Patient/donor demographics.

	Healthy Donor for Allogeneic Transplant (N = 68)	Patient Donor for Autologous Transplant (N = 94)
Age, years, median	45 (15–67)	Lymphoma: 48 (18–70) Multiple myeloma: 59 (34–71)
Sex	44 Male/24 Female	Lymphoma: 12 Male/22 Female Multiple myeloma: 32 Male/29 Female
Disease	N/A	Lymphoma: HL (11), NHL (23)
		Multiple myeloma: 60
Mobilization	G-CSF 10 mcg/kg/d × 5 days	Chemotherapy (disease-specific) and G-CSF 10 mcg/kg/d × 5 days
Days of collection	1	1
Total nucleated cells/kg collected (×10^8^/kg)	6.98 (0.86–18.9)	Lymphoma: 5.37 (1.7–11.7)
		Multiple myeloma: 2.59 (0.73–12.0)
CD34 ^+^ cells/kg collected (×10^6^/kg)	5.35 (2.4–12.8)	Lymphoma: 6.75 (1.9–34.6)
		Multiple myeloma: 5.98 (1.5–18.1)

**Table 2:** The relationship of percentage of inhibitory receptor-positive T cells in the grafts with percentage of inhibitory receptor ligand-positive M-MDSCs in the grafts.

Allograft		Correlation (R Value)	*p* Value
PD-L1^+^M-MDSC	PD-1^+^CD4^+^T	−0.192	0.126
PD-L1^+^M-MDSC	PD-1^+^CD8^+^T	−0.143	0.251
CD86^+^M-MDSC	CTLA-4^+^CD4^+^T	−0.388	0.001
CD86^+^M-MDSC	CTLA-4^+^CD8^+^T	−0.271	<0.05
Galectin-9^+^M-MDSC	TIM-3^+^CD4^+^T	−0.341	<0.01
Galectin-9^+^M-MDSC	TIM-3^+^CD8^+^T	−0.200	0.117
CD155^+^M-MDSC	TIGIT^+^CD4^+^T	−0.45	<0.0001
CD155^+^M-MDSC	TIGIT^+^CD8^+^T	−0.205	0.098
HVEM^+^M-MDSC	BTLA^+^CD4^+^T	−0.358	<0.01
HVEM^+^M-MDSC	BTLA^+^CD8^+^T	−0.327	<0.05
**Lymphoma autograft**			
PD-L1^+^M-MDSC	PD-1^+^CD4^+^T	−0.350	<0.05
PD-L1^+^M-MDSC	PD-1^+^CD8^+^T	−0.444	<0.01
CD86^+^M-MDSC	CTLA-4^+^CD4^+^T	−0.379	<0.05
CD86^+^M-MDSC	CTLA-4^+^CD8^+^T	−0.481	<0.01
Galectin-9^+^M-MDSC	TIM-3^+^CD4^+^T	−0.733	<0.001
Galectin-9^+^M-MDSC	TIM-3^+^CD8^+^T	−0.691	<0.001
CD155^+^M-MDSC	TIGIT^+^CD4^+^T	−0.648	<0.0001
CD155^+^M-MDSC	TIGIT^+^CD8^+^T	−0.440	<0.01
HVEM^+^M-MDSC	BTLA^+^CD4^+^T	−0.128	0.471
HVEM^+^M-MDSC	BTLA^+^CD8^+^T	−0.211	0.23
**MM autograft**			
PD-L1^+^M-MDSC	PD-1^+^CD4^+^T	−0.547	<0.0001
PD-L1^+^M-MDSC	PD-1^+^CD8^+^T	−0.531	<0.0001
CD86^+^M-MDSC	CTLA-4^+^CD4^+^T	−0.497	<0.0001
CD86^+^M-MDSC	CTLA-4^+^CD8^+^T	−0.295	<0.05
Galectin-9^+^M-MDSC	TIM-3^+^CD4^+^T	−0.524	<0.0001
Galectin-9^+^M-MDSC	TIM-3^+^CD8^+^T	−0.354	<0.01
CD155^+^M-MDSC	TIGIT^+^CD4^+^T	−0.597	<0.0001
CD155^+^M-MDSC	TIGIT^+^CD8^+^T	−0.528	<0.0001
HVEM^+^M-MDSC	BTLA^+^CD4^+^T	−0.359	<0.01
HVEM^+^M-MDSC	BTLA^+^CD8^+^T	−0.271	<0.05

## Data Availability

The raw data supporting the conclusions of this article are not publicly available due to privacy and ethical restrictions.

## Supplementary Materials

The following supporting information can be downloaded at: https://www.mdpi.com/article/10.3390/cells13181545/s1, Figure S1: Representative figure of effects of M-MDSC from lymphoma autograft on the PD-1 expression on autologous CD4^+^T cells; Figure S2: Representative Figure of effects of M-MDSC from allograft on the PD-1 expression on third party CD3^+^T cells.
