# Supplementary material for: The Interactions of T Cells with Myeloid-Derived Suppressor Cells in Peripheral Blood Stem Cell Grafts
Source: Cells. 2024 Sep 14;13(18):1545. doi: 10.3390/cells13181545 (PMC11429538; doi:10.3390/cells13181545)
Supplement: Supplementary file 1 [file cells-13-01545-s001.zip › cells-3170739-supplementary.pdf]

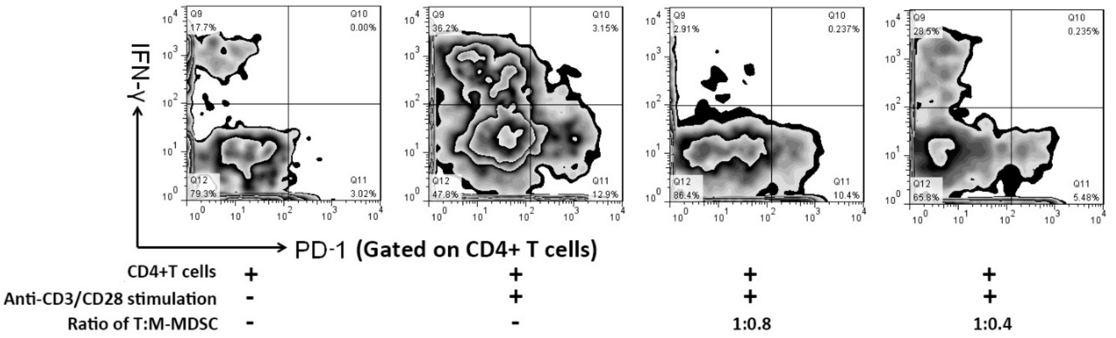

Supplementary Figure S1. Representative figure of effects of M-MDSC from lymphoma autograft on the PD-1 expression on autologous CD4$^+$T cells.

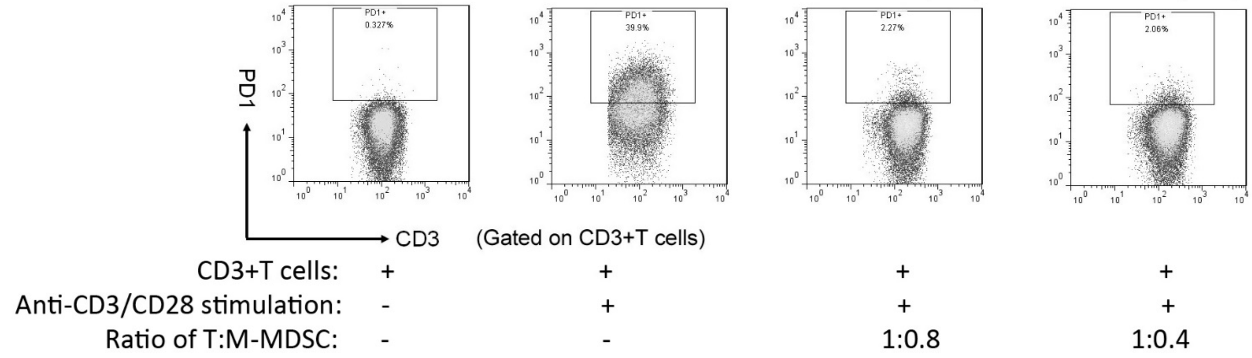

Supplementary Figure S2. Representative Figure of effects of M-MDSC from allograft on the PD-1 expression on third party CD3$^+$T cells
